# Supplementary material for: Covalent inhibition of the SARS-CoV-2 NiRAN domain via an active-site cysteine
Source: J Biol Chem. 2025 Mar 4;301(4):108378. doi: 10.1016/j.jbc.2025.108378 (PMC12013494; doi:10.1016/j.jbc.2025.108378)
Supplement: Supporting information [file mmc1.docx]

**Supporting information**

**Covalent inhibition of the SARS-CoV-2 NiRAN domain via an active-site cysteine**

Genaro Hernandez^1^, Adam Osinski^1,2^, Abir Majumdar^1,2^, Jennifer L. Eitson^3^, Monika Antczak^4^, Krzysztof Pawłowski ^1,2^, Hanspeter Niederstrasser^4^, Kelly A. Servage^1,2^, Bruce Posner^4^, John W. Schoggins^3^, Joseph M. Ready^4^, Vincent S. Tagliabracci*^1,2,5,6^

**Affiliations:**

^1^Department of Molecular Biology, University of Texas Southwestern Medical Center, Dallas, Texas 75390, USA.

^2^Howard Hughes Medical Institute, University of Texas Southwestern Medical Center, Dallas, Texas 75390, USA

^3^Department of Microbiology, University of Texas Southwestern Medical Center, Dallas, Texas 75390, USA.

^4^Department of Biochemistry, University of Texas Southwestern Medical Center, Dallas, Texas 75390, USA.

^5^Harold C. Simmons Comprehensive Cancer Center, UT Southwestern Medical Center, Dallas, Texas 75390, USA

^6^Hamon Center for Regenerative Science and Medicine, University of Texas Southwestern Medical Center, Dallas, Texas 75390, USA

*Correspondence to: Vincent S. Tagliabracci

**Email:** [vincent.tagliabracci@utsouthwestern.edu](mailto:vincent.tagliabracci@utsouthwestern.edu)

**Keywords:** NiRAN, RNA capping, pseudokinase, SARS-CoV-2, COVID-19, enzyme inhibitor, inhibitor, drug screening, drug discovery, enzyme structure

**Running title:** Covalent inhibition of the SARS-CoV-2 NiRAN domain

**Supplemental Figures**

**
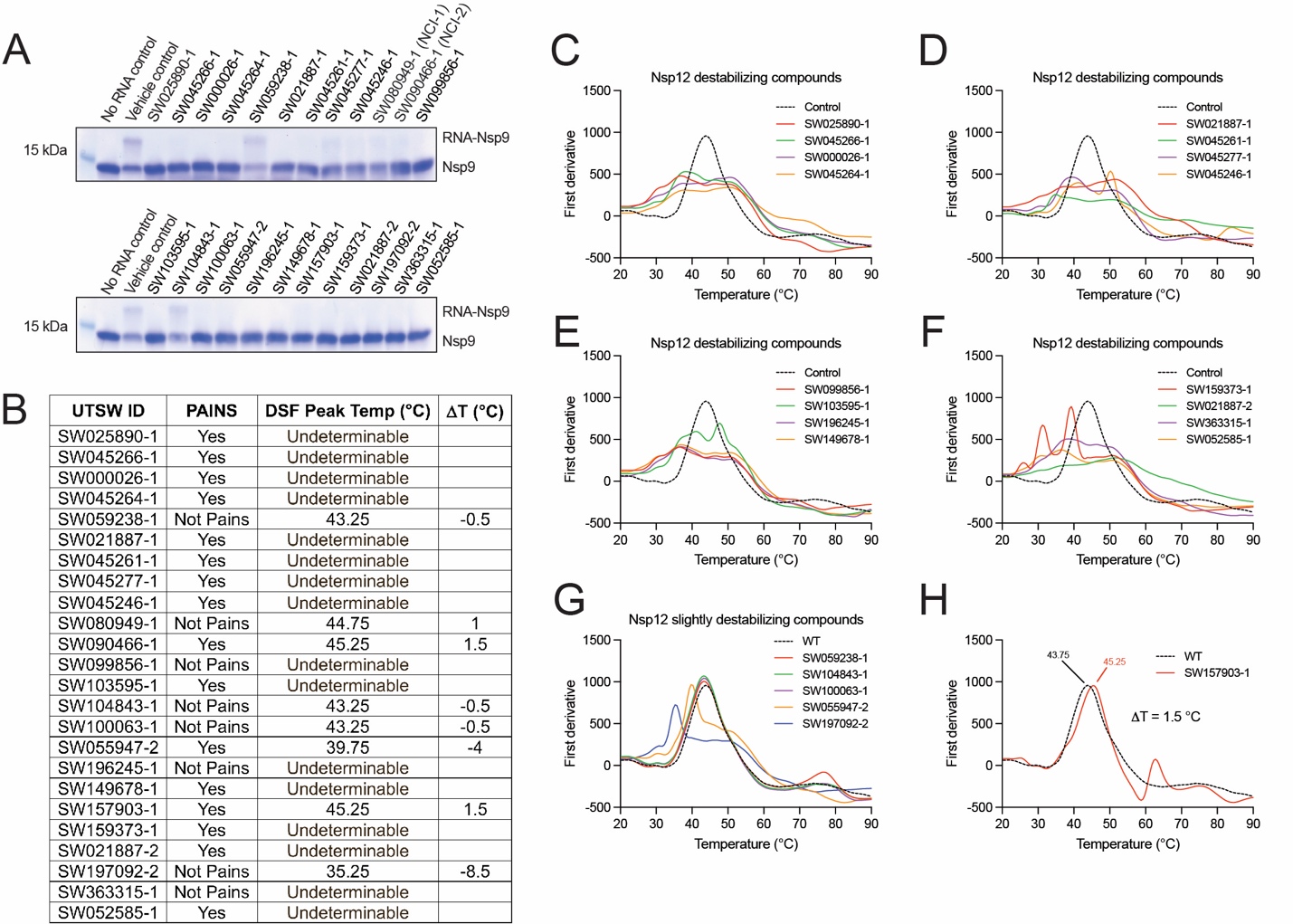
**

**Figure S1. Characterization of the small molecule identified in the HTS. A,** RNAylation activity of nsp12, (500 nM) following incubation with various small molecules (20 μM) identified in the HTS. Reactions were initiated with 100 μM of SARS-CoV-2 genomic leader sequence corresponding to the first 10 bases in the genome, and the reaction products were analyzed by SDS-PAGE and Coomassie staining. B, table depicting the ID, PAINS class, and thermal stability profile (peak temp. and change melting temperature; ΔT) of the small molecules identified from the HTS. C-H, differential scanning fluorimetry profiles of nsp12 (10 µM) in the presence of the small molecules identified from the HTS. Compounds were grouped into nsp12 destabilizing compounds (C-F), nsp12 slightly destabilizing compounds (G) and SW157903 (H), which stabilized nsp12; however, we did not follow up on due to its PAINS class.


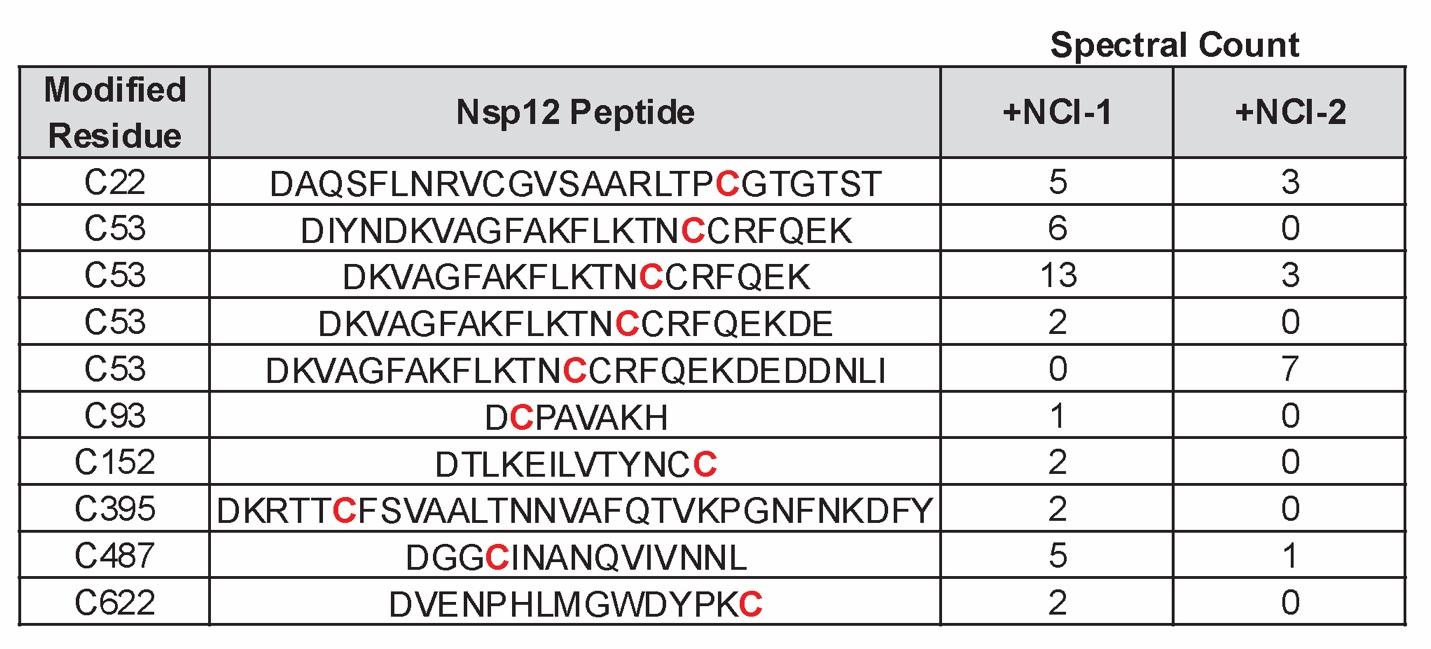


**Figure S2. LC-MS/MS analysis of nsp12 bound to NCI-1 and NCI-2.** Table depicting the nsp12 peptides identified by LC-MS/MS to be covalently modified by NCI-1 (+192 Da) or NCI-2 (+205 Da) on cysteine residues. Modified cysteines are highlighted in red. Spectral counts reflect the number of MS2 peptide spectral matches as determined by Mascot software searches.


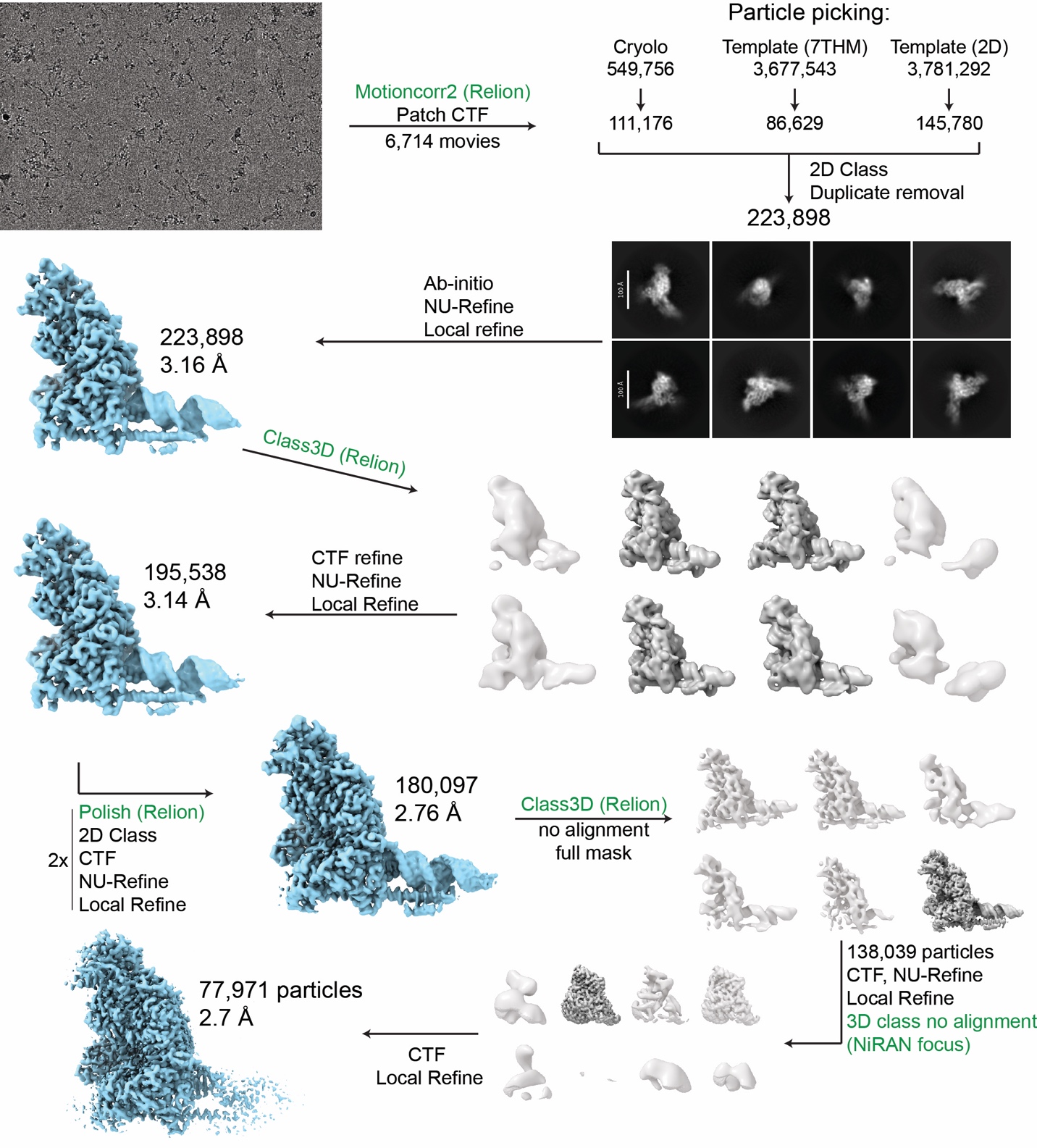


**Figure S3. Cryo-EM data processing scheme.** Data was processed in CryoSPARC 4.2.1. Steps indicated in green were performed in Relion 4. Particles were picked with CryoSPARC and crYOLO 1.9.3.


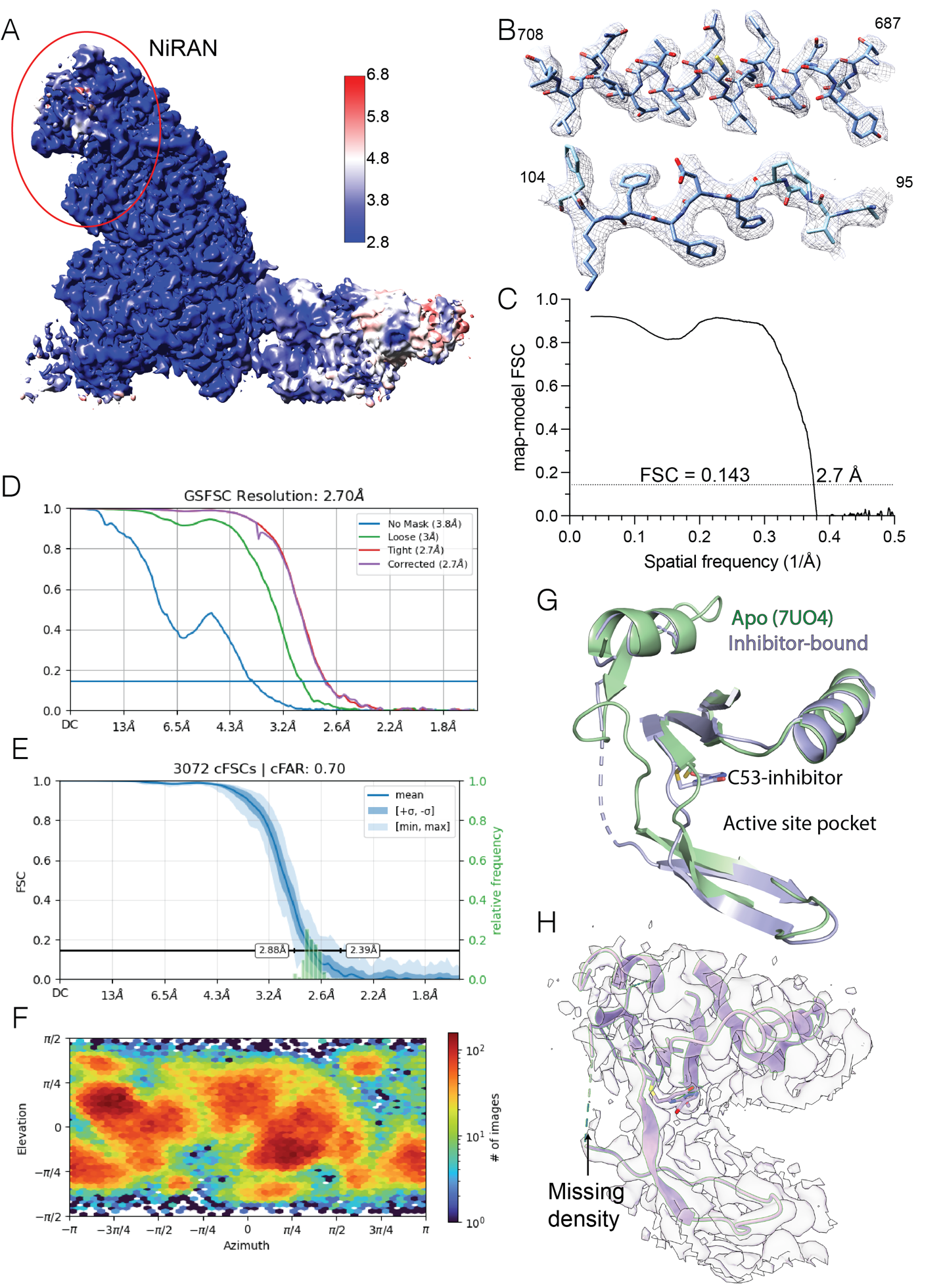


**Figure S4. Cryo-EM structure quality metrics.** A, local resolution estimation of the RTC bound to NCI-2. NiRAN domain is indicated. B, example coulombic map density and model fit within the polymerase domain (top) and the NiRAN domain (bottom). C, map-model FSC curve of the RTC bound to NCI-2. D, gold-standard FSC curves for the final refinement. E. The conical FSC Area Ratio plot. Reported Sampling Compensation Factor (SCF) was 0.902. F, particle angular distribution plot for the final refinement. G. Binding to the NCI-2 destabilizes a strand proximal to Cys53. The missing structure is presented as dashed lines on the left side of the panel. Light-blue cartoon of the inhibitor-bound structure is aligned with an apo structure in green; pdb: 7UO4 (35). H. A view of NiRAN structure with the missing density around the inhibitor binding site.
